# The Treatment of a Multisubunit Defect of the Earlobe Involving an Exposed Parotid Gland

**DOI:** 10.7759/cureus.50224

**Published:** 2023-12-09

**Authors:** Harib H Ezaldein

**Affiliations:** 1 Mohs Micrographic Surgery, Bennett Surgery Center, Santa Monica, USA; 2 Dermatology, Miami Dermatology and Mohs Surgery, Miami, USA

**Keywords:** high risk skin cancer, plastic reconstruction, mohs surgery, melanoma, rhombic flap

## Abstract

Defects with multiple aesthetic subunits may need specific approaches for each subunit. We present a case of a post-surgical defect in a patient who underwent Mohs micrographic surgery for an invasive melanoma of the earlobe with an exposed parotid gland. We utilized a retroauricular-based rhomboid flap to provide full and immediate coverage for earlobe reconstruction in the setting of insufficient infra-auricular recruitable skin. The addition of Z-plasties at the base of an interpolation flap may reduce rotational restraint, thereby improving perfusion while enhancing flap extension for complete wound coverage where there is a lack of abundant donor tissue. The patient healed appropriately with no evidence of tumor recurrence.

## Introduction

Mohs micrographic surgery, a highly specialized and precise technique originally developed for the treatment of non-melanoma skin cancers, has emerged as a valuable tool in the management of melanoma [1]. While melanoma is a complex and potentially aggressive form of skin cancer, Mohs surgery offers a unique approach that goes beyond traditional excision. Its distinctive advantage lies in its ability to provide not only microscopic control over tumor removal but also in identifying melanomas with extensions that are often invisible to the naked eye. The significance of Mohs micrographic surgery for melanoma patients cannot be overstated. Traditional excisions may leave behind subtle, unnoticed extensions of the tumor, which can necessitate additional surgeries and lead to an increased risk of recurrence. In contrast, Mohs surgery, with its meticulous, stage-by-stage hematoxylin and eosin frozen section analysis of the removed tissue, ensures that the entire tumor is eliminated, even when its spread is not apparent in standard clinical examination [2-3].

This case report will delve into the unique reconstructive challenges of Mohs micrographic surgery in the context of melanoma management, highlighting how this method revolutionizes the treatment approach for this often unpredictable and aggressive skin cancer. We illustrate the principle of earlobe reconstruction for surgical defects resulting from microscopically controlled invasive melanoma removal.

## Case presentation

**Figure 1:**
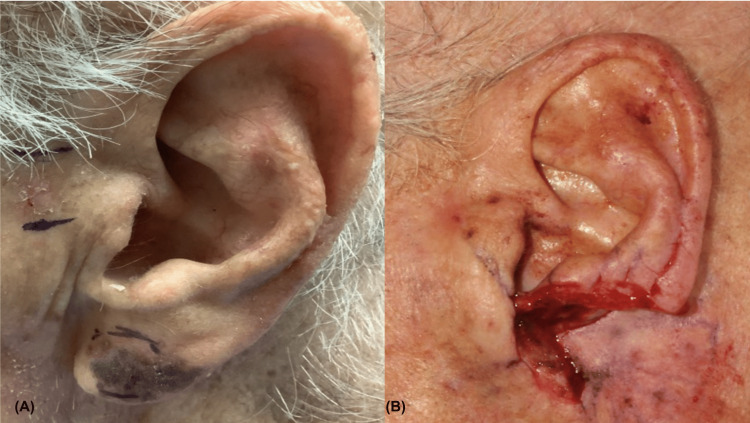
(A) Anterolateral view of preoperative melanoma lesion, (B) followed by multisubunit postoperative defect, measuring 6.1 cm x 5.3 cm and extending to cartilage and parotid gland

A 92-year-old man presented with a lentigo maligna melanoma, appearing as a 2.6 cm x 1.8 cm irregular variegated patch on the left anterior earlobe (Figure 1).

**Figure 2:**
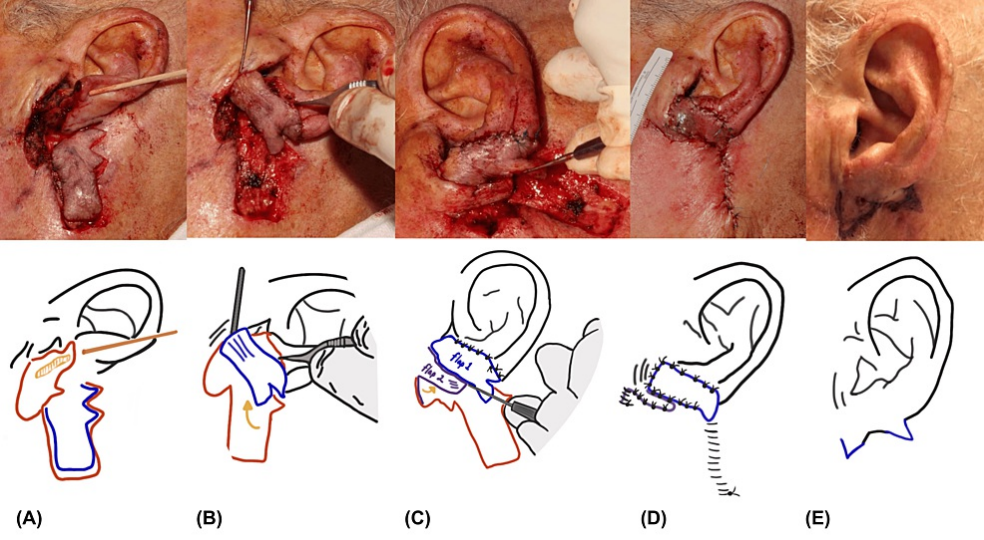
(A-B) Surgical repair of the multisubunit defect. A rhombic flap with double Z-plasty is rotated superiorly for anterior lobule and helical coverage. (C) A second rhombic flap is transposed into the lateral cheek aspect of the defect to cover the parotid. (D) Secondary defects were closed primarily. (E) The attached pedicles and cicatricial contour were revised with V-Y plasty at four weeks Credits: Harib H. Ezaldein, MD

The tumor was removed with micrographic surgery in four stages. The resultant surgical defect measured 6.1 cm x 5.3 cm, involved multiple cosmetic subunits, extended to adipose tissue, and exposed cartilage and superficial parotid gland (Figure 2). A mastoid skin-based interpolation was considered, though its wide pedicle base would present a higher risk for bleeding complications in our elderly patient. To narrow the pedicle, enhance flap movement, and provide coverage with a reliable blood supply, we ultimately chose a posterior-based interpolation flap with the addition of a double Z-plasty. To create the interpolated rhombic flap, incisions of equal length were made medial and inferior to the mastoid process (Figure 2).

Two Z-plasties were added laterally to the base of the rhombic flap to provide enhanced flap movement at angles of 120 and 90 degrees respectively. We then rotated, advanced, and transposed the flap into the defect and secured it into place with 4-0 polyglycolic acid sutures and 4-0 polypropylene sutures (Figure 2). The lateral cheek and parotid defect components were repaired with a traditional rhombic flap, while secondary and tertiary defects from the flap were repaired primarily. Four weeks later, the flap was divided at the base to free the lobule.

**Figure 3:**
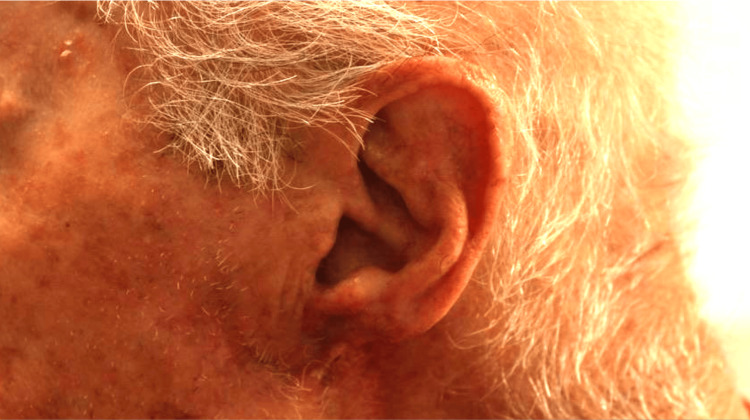
Seven-month postoperative appearance of the surgical site

When seen at the seven-month follow-up (Figure 3), the flap had blended in well with the surrounding skin and provided acceptable helical and lobular contour, all without any evidence of tumor recurrence.

## Discussion

This defect contains multiple subunits such as the conchal bowl, antihelical fold, lower helix, and earlobe. It also affects the infra-auricular and lateral cheek skin, with exposed helical cartilage and parotid gland. An ideal approach involves repairing individual subunits and minimizing free margin distortion or postoperative complications.

Granulation or healing with secondary intent is not appropriate considering the risk of parotid gland fistulae, blurring of cosmetic subunit boundaries, and auricular contour distortion. A partial closure of certain subunits while granulating others also carries the same cosmetic and fistula risks. Split- or full-thickness skin grafts for each cosmetic subunit are a viable option, although lobule reconstruction cannot reliably be performed with this approach due to risks of graft contraction and necrosis. The lack of medial and infralobular skin precludes transposition flaps for earlobe construction, as done in some reports [4,5]. A single-stage Gavello approach with a second flap for the preauricular cheek defect was considered, but there was insufficient recruitable skin [6,7].

The random pattern flap is formed in the retroauricular inframedial mastoid skin and receives a reliable blood supply from arcades of the occipital branch of the posterior auricular artery, located horizontally behind the ear. A rhombic flap with double Z-plasties reduces rotational restraint, and the width of the pedicle base enhances flap movement and provides a predictable vascular supply.

## Conclusions

In cases involving defects with multiple cosmetic subunits, it is crucial to consider tailored approaches for each subunit to achieve optimal aesthetic outcomes. For earlobe reconstruction, retroauricular-based flaps offer a valuable solution when there is a shortage of recruitable skin in the infra-auricular region. These flaps can provide comprehensive and immediate coverage, addressing the specific needs of earlobe reconstruction effectively. To enhance flap extension and ensure complete wound coverage in situations where abundant donor tissue is lacking, the incorporation of Z-plasties at the base of an interpolation flap is a valuable technique. This approach can reduce rotational restraint, leading to improved perfusion and overall flap performance.
